# Complete Genome Sequence of *Pandoraea pnomenusa* 3kgm, a Quorum-Sensing Strain Isolated from a Former Landfill Site

**DOI:** 10.1128/genomeA.00427-14

**Published:** 2014-05-08

**Authors:** Kok-Gan Chan, Wai-Fong Yin, Share-Yuan Goh

**Affiliations:** Division of Genetics and Molecular Biology, Institute of Biological Sciences, Faculty of Science, University of Malaya, Kuala Lumpur, Malaysia

## Abstract

*Pandoraea pnomenusa* strain 3kgm has been identified as a quorum-sensing strain isolated from soil. Here, we report the complete genome sequence of *P. pnomenusa* strain 3kgm by using the Pacific Biosciences single-molecule real-time (PacBio RS SMRT) sequencer high-resolution technology.

## GENOME ANNOUNCEMENT

*Pandoraea pnomenusa* strain 3kgm was isolated from a soil sample obtained from an ex-landfill site in Puchong, Malaysia. *Pandoraea* spp. are closely related to and are commonly misidentified as *Ralstonia* spp. or belonging to the *Burkholderia cepacia* complex. To accurately identify the organism to the genus and species level, 16S rRNA gene-based PCR assays (1), next-generation sequencing, and matrix-assisted laser desorption ionization–time of flight (MALDI-TOF) mass spectrometry (2) were used. The availability of the complete genome of *P. pnomenusa* strain 3kgm will facilitate research for this strain, as it provides a fundamental molecular evolution study of its genetic foundation especially in clinical microbiology and to avoid the misidentification of a *Pandoraea* sp.

In the world of unicellular bacteria, signal integration from the bacterial phenotype and bacterial environment form a network of cellular transduction mechanisms to control their gene expression (3). Using *lux*, *gfp*, or *lacZ* acyl homoserine lactone (AHL) biosensor reporter gene fusions or pigment induction, numerous AHL biosensor assays have been developed to facilitate the screening of AHL production (4). The positive quorum-sensing activity of *P. pnomenusa* strain 3kgm was screened by the AHL biosensor of *Chromobacterium violaceum* and *Escherichia coli* [pSB 401] (5, 6).

The PacBio single-molecule real-time (RS SMRT) sequencer is a third-generation sequencing technology with no amplification required (7). By using this PacBio RS SMRT technology and a low-input 10-kb library preparation, the strain 3kgm genome was sequenced and found to be 5,429,297 bp long with 64.72% G+C content in 1 contig, a consensus accuracy of 99.9997%, and 189.56-fold coverage of the genome. The Hierarchical Genome Assembly Process (HGAP) assembler and targeted resequencing pipeline provided by PacBio in the SMRT Portal were employed to derive this single-contig complete closed genome. HGAP consists of preassembly, *de novo* assembly with Celera Assembler, and assembly polishing with Quiver. Before assembly using Celera assembler (CA) version 7.0 software, the PacBio Rs_PreAssembler.1 module with default minimum subread length of 500 bp, a minimum read quality of 0.80, and a minimum subread length of 5,000 bp was used to perform error correction of the PacBio RS-generated raw reads. The initial genome assembly was further refined through the PacBio RS_Resequencing.1 software (8). With this refined closed genome sequence, gene prediction was performed through PROkaryotic Dynamic programming Gene-finding ALgorithm (Prodigal) (version 2.60) (9), while rRNA genes were predicted with RNAmmer (10) and tRNA genes were predicted with tRNAscan-SE (11). Subsequently, it was annotated with BLASTx against the NCBI-nt/nr updated database and UniProt database (12, 13). Gene prediction resulted in 4,850 open reading frames (ORFs), and a copy each of 5S rRNA, 16S rRNA, 23S rRNA, and tRNA genes were identified.

### Nucleotide sequence accession number.

This whole-genome shotgun project has been deposited at DDBJ/EMBL/GenBank under the accession no. CP006900. The version described in this paper is the first version.
